# Hypoxia alters the recruitment of tropomyosins into the actin stress fibres of neuroblastoma cells

**DOI:** 10.1186/s12885-015-1741-8

**Published:** 2015-10-16

**Authors:** Joshua J. Glass, Phoebe A. Phillips, Peter W. Gunning, Justine R. Stehn

**Affiliations:** Oncology Research Unit, School of Medical Sciences, UNSW Australia, Room 229, Wallace Wurth Building, Sydney, NSW 2052 Australia; Pancreatic Cancer Translational Research Group, Lowy Cancer Research Centre, UNSW Australia, Sydney, NSW 2052 Australia; Current address: ARC Centre of Excellence in Convergent Bio-Nano Science and Technology, and Department of Microbiology and Immunology, The University of Melbourne, at the Peter Doherty Institute for Infection and Immunity, Melbourne, VIC 3010 Australia

**Keywords:** Hypoxia, Actin, Tropomyosin, Neuroblastoma

## Abstract

**Background:**

Neuroblastoma is the most common extracranial solid tumor of childhood. The heterogeneous microenvironment of solid tumors contains hypoxic regions associated with poor prognosis and chemoresistance. Hypoxia implicates the actin cytoskeleton through its essential roles in motility, invasion and proliferation. However, hypoxia-induced changes in the actin cytoskeleton have only recently been observed in human cells. Tropomyosins are key regulators of the actin cytoskeleton and we hypothesized that tropomyosins may mediate hypoxic phenotypes.

**Methods:**

Neuroblastoma (SH-EP) cells were incubated ± hypoxia (1 % O_2_, 5 % CO_2_) for up to 144 h, before examining the cytoskeleton by confocal microscopy and Western blotting.

**Results:**

Hypoxic cells were characterized by a more organized actin cytoskeleton and a reduced ability to degrade gelatin substrates. Hypoxia significantly increased mean actin filament bundle width (72 h) and actin filament length (72–96 h). This correlated with increased hypoxic expression and filamentous organization of stabilizing tropomyosins Tm1 and Tm2. However, isoform specific changes in tropomyosin expression were more evident at 96 h.

**Conclusions:**

This study demonstrates hypoxia-induced changes in the recruitment of high molecular weight tropomyosins into the actin stress fibres of a human cancer. While hypoxia induced clear changes in actin organization compared with parallel normoxic cultures of neuroblastoma, the precise role of tropomyosins in this hypoxic actin reorganization remains to be determined.

**Electronic supplementary material:**

The online version of this article (doi:10.1186/s12885-015-1741-8) contains supplementary material, which is available to authorized users.

## Background

Neuroblastoma is the most common extracranial solid tumor of childhood. These neoplasms derive from immature cells of the sympathetic nervous system (SNS) and can present at any SNS structure, most commonly in and around the adrenal glands [1, 2]. Over 90 % of patients are younger than 5 years at diagnosis and over half present with metastatic spread, predominately to the bone marrow and bone [3, 4]. While overall survival for stage 1 and 2 patients is 96.2 % and 88.6 %, respectively, overall survival for high-grade, stage 4 patients remains low at 22.4 % [5].

Solid tumors are heterogeneous, complex structures that must be analysed in the context of their microenvironment [6]. Structurally and functionally poor quality tumor vasculature leads to regions of low oxygen (O_2_) perfusion referred to as hypoxia [7]. Prognoses are made worse by hypoxic microenvironments, which create genetic instability fundamental to tumor progression [8] and increase neuroblastoma resistance to radiotherapy and standard chemotherapies [9–12]. The actin cytoskeleton is essential for various hypoxic phenotypes, including altered motility and invasion. However, until now, no studies have examined the effect of hypoxia on the actin cytoskeleton of neuroblastoma.

The aggressive hypoxic phenotype of neuroblastoma is well documented. Over a decade ago, Ginis and Faller [13] observed that Kelly neuroblastoma cells increased their invasiveness and decreased their adhesion to endothelium when treated to hypoxic conditions,, indicating a pro-metastatic phenotype. Hypoxia-fostered malignancies are worsened by neuroblastoma dedifferentiation *in vitro* and *in vivo*, with reversion to an immature and neural crest-like phenotype [14]. Such dedifferentiation is associated with increased tumor heterogeneity and aggressiveness [14, 15]. Moreover, the neuroblastoma transcriptome and proteome are dramatically altered by hypoxia toward malignant and metastatic profiles. Hypoxia upregulates the expression of genes associated with growth, survival and drug resistance [16] and induces a pro-metastatic gene program [17].

Hypoxia-inducible factor (HIF) transcription factors are believed to be the master transcriptional regulators of the hypoxic response [18, 19]. HIFs are heterodimeric transcription factors composed of an O_2_-regulated α subunit and a constitutively expressed β subunit [20]. Under conventional tissue culture O_2_ tensions (20 % O_2_), referred to as normoxia, HIF-α subunits are rapidly hydroxylated, ubiquitinated and proteasomally degraded [21–23]. At 1 % O_2_, HIF-1α and HIF-2α subunits are stabilized and translocate to the nucleus [24]. Whether caused by hypoxia or oncogenic mutations, increased HIF levels are largely associated with poor prognoses in a variety of cancers [18]. In neuroblastoma, HIF-2α predicts poor patient outcome, while HIF-1α has been associated with a favorable prognosis [25]. Intriguingly, hypoxia-induced chemoresistance is HIF-1α-dependent [11, 12]. More recently, the sonic hedgehog signaling pathway has been implicated in HIF-1α-mediated proliferation and invasion of neuroblastoma cells [26].

Actin is a core component of the eukaryotic cytoskeleton. It is well established that changes in actin organization and the levels of its binding proteins are essential to the cancer cell phenotype (reviewed in [27, 28]). In fact, the actin cytoskeleton is essential for a variety of processes hijacked or subverted by hypoxic cancer cells. These include proliferation, invasion, motility, adhesion and apoptosis [28–32]. A recent study found that hypoxia led to HIF-1α-dependent actin filament rearrangement in mouse L929 fibrosarcoma cells [33]. However, to our knowledge, no studies have previously examined the role of the actin cytoskeleton in hypoxic human cancer phenotypes.

The tropomyosin family of proteins are involved in most, if not all, actin cytoskeletal functions [34]. Tropomyosins exist as rod-like coiled coil dimers that form head-to-tail polymers [35] and wrap along the major grooves of most actin filaments. Four genes, TPM1-4, encode over 40 mammalian isoforms through splicing and alternative promoters. High (HMW) and low molecular weight (LMW) isoforms correspond to ~284 and 247 amino acids, respectively [36]. Tropomyosins contribute to the spatial and temporal regulation of the actin cytoskeleton in an isoform-specific manner, by regulating actin’s association with a plethora of actin-binding proteins [34]. Interestingly, tropomyosins are implicated in the pathogenesis of cancer. HMW isoforms are consistently down-regulated in transformed cells, while malignant cells display an increased reliance on LMW isoforms [37]. We have previously observed consistent down-regulation of HMW isoforms (Tm1, Tm2 and Tm3) and an increased reliance on LMW isoforms (Tm5NM1/2 and Tm4) in all profiled neuroblastoma and melanoma cell lines, as well as transformed primary BJ fibroblasts [38].

We hypothesized that tropomyosins may facilitate the hypoxic phenotypes of cancers such as neuroblastoma by driving changes in the actin cytoskeleton. We therefore aimed (1) to characterize the hypoxic phenotype by observing changes in neuroblastoma cell proliferation and invasion, and (2) to examine hypoxia-induced changes in the actin cytoskeleton, including tropomyosin isoform expression and localization.

In this study we have demonstrated hypoxia-induced changes in tropomyosin expression and localization in a human cancer. Changes in actin organization characteristic of reversion of the transformed phenotype are induced by hypoxia at 72 h in neuroblastoma. This correlates with expected isoform-specific changes in tropomyosin localization. However, isoform specific changes in tropomyosin expression are more evident at 96 h. Hypoxia induces clear changes in actin organization compared with parallel normoxic cultures of neuroblastoma. However, the role tropomyosins might play in driving this hypoxic actin reorganization remains to be further elucidated.

## Methods

### Cell culture

SK-N-SH-EP (SH-EP) human neuroblastoma cells [39, 40] were a generous gift from Children’s Hospital Westmead. STR fingerprinting was performed to confirm the cell line’s identity. SH-EP were maintained in growth media containing Dulbecco’s modified Eagle’s medium (DMEM)/high glucose (4.5 g/L), L-glutamine (4.0 mM) and sodium pyruvate (4.0 mM) (HyClone-Thermo Scientific, UT, USA), supplemented with 10 % v/v fetal bovine serum (FBS) (Gibco-Life Technologies, NY, USA). Cells maintained at 37 °C in humidified, normoxic 5 % CO_2_/95 % air incubator (~20 % O_2_). All replicate experiments conducted within 6 passages. All studies reported in this manuscript were *in vitro* cell based studies using human cancer cell lines that are commercially available.

### Hypoxic incubation

In neuroblastoma, metabolic hypoxia occurs below 8–10 mmHg O_2_ (approx. 1.1–1.3 % O_2_) [41]. To induce hypoxia, cells were placed inside a modular incubator chamber (Billups-Rothenberg, CA, USA) and flushed with 1 % O_2_/5 % CO_2_/94 % N_2_ gas (BOC Australia, NSW, AUS) for 8 mins at 25 L/min. The sealed chamber was incubated at 37 °C and flushing was repeated every 24 h.

### Cell proliferation

Cells were seeded in 100 mm plates (Costar-Corning, NY, USA) at 9.2 × 10^4^/10 mL media and incubated at 37 °C overnight, before incubating ± hypoxia for 24–144 h. Cells were harvested with trypsin-EDTA (Gibco-Life Technologies, NY, USA) and resuspended in growth media. Live cells counted using Countess® Automated Cell Counter after mixing 1:1 with 0.4 % w/v trypan blue (Invitrogen, CA, USA).

### Invasion assays

QCM Gelatin Invadopodia Assay (Millipore, MA, USA) performed as per manufacturer’s instructions in 8-well Lab-Tek® chamber slides (Nunc, IL, USA). Briefly, cells seeded at 1.6 × 10^4^/well onto GFP-tagged gelatin to examine invadopodial matrix-degradation. After 72–96 h ± hypoxia, cells were fixed and stained with kit-supplied DAPI nucleic acid stain and filamentous actin-binding TRITC-phalloidin. Coverslips mounted with ProLong Gold Antifade Reagent (Invitrogen, OR, USA) and cells visualized using an Axioskop 40 epifluorescent microscope (20× objective) (Zeiss, Göttingen, Germany). Five fields of view obtained per condition. Gelatin degradation, cell area and cell counts quantified using ImageJ (V1.46; NIH).

### Actin cytoskeleton and tropomyosin organization

Cells seeded at 9.2 × 10^3^/mL media on coverslips (Carl Zeiss Microscopy, NY, USA) and incubated overnight in normoxia. Cells then incubated ± hypoxia for 48–96 h (actin cytoskeleton) or 72 h (tropomyosin). Cells fixed in 4 % w/v paraformaldehyde (PFA) in phosphate-buffered saline (PBS) for 15 mins, then washed thrice in PBS. All staining performed at room temperature (RT), as below.

### Actin and tropomyosin immunofluorescence staining

For actin filament staining, cells were permeabilized with 0.1 % v/v TritonX-100 for 5 mins, washed thrice in PBS, blocked in 0.5 % w/v bovine serum albumin (BSA) in PBS for 1 h, incubated with TRITC-phalloidin (1:1,000; Sigma-Aldrich) in 0.5 % w/v BSA and washed thrice in PBS. For anti-tropomyosin staining, cells were permeabilized with −80 °C methanol for 15 mins, washed thrice in PBS, blocked with 2 % v/v FBS in PBS for 30 mins, incubated with primary tropomyosin isoform-specific antibody for 2 h diluted in 2 % v/v FBS as per Table 1, washed thrice in PBS, incubated with appropriate Alexa555- or Alexa488-conjugated secondary antibody for 1 h in the dark, diluted in 2 % v/v FBS as per Table 1, and washed thrice in PBS. All coverslips then incubated with DAPI nucleic acid stain (1:10,000) in PBS for 1 min, washed thrice in PBS and mounted onto microscope slides using ProLong Gold Antifade Reagent.

**Table 1:** Primary and secondary antibodies

Name	1° /2°	Target	Dilution	Species	Commercial availability	Ref.
α/9d	1°	Tm1, 2, 3, 5a*, 5b*, 6*	WB/IF: 1:500	Mouse monoclonal (IgG2b κ; clone 15D12.2	–	[79]
γ/9d	1°	Tm5NM1/2	WB/IF: 1:500	Mouse monoclonal (IgG2b κ; clone 2G10.2)	–	[79]
WD4/9d	1°	Tm4	WB/IF: 1:500	Rabbit polyclonal	Millipore	[80]
CG1	1°	Tm1	IF: 1:50	Mouse monoclonal (IgG)	–	[79]
CG3	1°	Tm5NM1-11	WB/IF: 1:150	Mouse monoclonal (IgM)	–	[79]
CGβ6	1°	Tm2, 3	IF: 1:150	Mouse monoclonal (IgM)	–	[79]
Tm311	1°	Tm1, 2, 3, 6*, Br1*, plus α-,β-, γ-muscle*	IF: 1:500	Mouse monoclonal (IgG1), clone TM311	Sigma-Aldrich	[81]
C4 Actin	1°	Actin	WB: 1:5000	Mouse monoclonal, (IgG1κ; clone C4)	Millipore	[82]
α-actinin	1°	α-actinin 1-4	WB: 1:200	Rabbit polyclonal (H-300)	Santa Cruz Biotechnology	[83]
HIF-1α	1°	HIF-1α	WB: 1:500	Mouse monoclonal (IgG1; clone 54)	BD Biosciences	[20]
HIF-2α	1°	HIF-2α	WB: 1:500	Rabbit polyclonal	Novus Biologicals	[84]
GAM-HRP	2°	Mouse (H + L)	WB: 1:5000	Goat polyclonal	Bio-Rad	[85]
GAR-HRP	2°	Rabbit (H + L)	WB: 1:5000	Goat polyclonal	Bio-Rad	[85]
Alexa Fluor® 555 GAM	2°	Mouse (H + L)	IF: 1:1000	Goat polyclonal	Molecular Probes	[86]
Alexa Fluor® 488 GAR	2°	Rabbit (H + L)	IF: 1:1000	Goat polyclonal	Molecular Probes	[86]

*Tropomyosin isoforms not expressed by SH-EP (Stehn et al., unpublished data). Those antibodies not commercially available were generated in-house

*WB* Western blot, *IF* immunofluorescence, *1°* primary antibody, *2°* secondary antibody, *GAM* goat anti-mouse, *GAR* goat anti-rabbit, *HRP* horseradish peroxidase, *H* heavy chain of IgG, *L* light chain of IgG

Single z-plane images obtained using an SP5 2P STED confocal microscope (40× oil objective) (Leica Microsystems, Wetzlar, Germany). Actin filament bundle width and length were quantitated using a linear-feature detection algorithm developed in collaboration with the CSIRO and previously described [42].

### Protein expression analysis

Cells were seeded in 100 mm plates at 9.2 × 10^4^/10 mL, incubated overnight in normoxia at 37 °C, before incubating ± hypoxia for 48–144 h. Cells were harvested using trypsin-EDTA, pelleted by centrifugation (1,200 rpm, 4 °C, 10 mins) and stored at −80 °C unless used immediately. Cells lysed in 100 μl/4 × 10^5^ cells of radioimmunoprecipitation assay (RIPA) buffer (6.67 mL 1.5 M Tris pH 8.0, 2 mL NP-40, 1 g deoxycholic acid, 1 mL 20 % w/v SDS, 1.752 g NaCl made to 200 mL with ddH_2_O) containing complete protease inhibitor cocktail (Roche Applied Science, IN, USA). For HIF-1/2α immunoblots, plates were transferred immediately to ice, rinsed with ice-cold PBS containing complete protease inhibitor cocktail and mechanically scraped using 50–100 μl RIPA buffer containing protease inhibitor cocktail. All lysates incubated on ice for 20 mins before centrifugation (16,100 × g, 4 °C, 10 mins). Supernatants transferred to new tubes. Total protein concentration estimated using bicinchoninic acid (BCA) protein assay (Thermo Scientific, IL, USA) and/or Direct Detect™ Spectrometer (EMD Millipore Corporation, MA, USA), as per manufacturers’ instructions.

### Western blotting

Lysates mixed with 4× Laemmli sample buffer (62.5 mM Tris–HCl pH 6.8, 10 % v/v glycerol, 1 % v/v SDS, 0.005 % w/v bromophenol blue, 355 mM 2-mercaptoethanol) in PBS to give total protein/lane of 6–10 μg (tropomyosin) or 25–35 μg (HIF-1α/2α). Samples heated for 5 mins at 95 °C and loaded onto 12.5 % (tropomyosin) or 8 % (HIF-1/2α) v/v SDS-PAGE gels with Precision Plus Protein™ standards (Bio-Rad, CA, USA). Gel electrophoresis performed in running buffer (1.0 L milli-Q H_2_O, 2.9 g Tris, 14.5 g glycine, 1 g SDS) at 120 V (Mini-PROTEAN® Tetra Cell; Bio-Rad, CA, USA). Proteins transferred to Immobilon-P polyvinylidene difluoride (PVDF) membranes (Millipore, MA, USA) in transfer buffer (1.6 L milli-Q H_2_O, 400 mL methanol, 5.8 g Tris, 29 g glycine) for 2 h at 80 V on ice.

Membranes blocked in 5 % w/v skim milk (SM) in Tris-buffered saline containing 0.1 % v/v Tween-20 (TBST) for 30 mins at RT. Membranes then incubated with primary antibody diluted as per Table 1 in 2 % w/v SM/TBST for 2 h at RT with constant agitation (tropomyosin and actin) or in 5 % w/v SM/TBST overnight at 4 °C (HIF-1/2α). Membranes washed thrice in TBST and incubated with appropriate HRP-conjugated secondary antibody (Table 1) in 2 % v/v SM/TBST for 1 h at RT. Membranes washed thrice in TBST, incubated with enhanced chemiluminescence (ECL) reagents (GE Healthcare, Amersham, UK) and visualized using medical radiographic film (Fuji Medical, Tokyo, Japan) or ImageQuant™ LAS-4000 (GE Healthcare, Munich, Germany). Densitometry performed on ImageJ. All results normalized to C4 actin loading control.

### Statistical analysis

Statistical analysis was conducted using two-sided t-tests, or two-way ANOVA when testing three or more means (GraphPad Prism V6.0). Results are mean ± SEM. *P* <0.05 was considered statistically significant.

## Results

### Hypoxia increases the levels of HIF-1α and HIF-2α in SH-EP neuroblastoma cells

HIF-1α and HIF-2α are commonly used markers of cellular hypoxia and these were used to confirm induction of hypoxia. Expression levels of both HIF-1α and HIF-2α increased as early as 8 h following hypoxic incubation compared with normoxic control cells (Additional file 1: Figure S1). HIF-1α levels increased from 8 to 144 h hypoxia, while hypoxia-induced HIF-2α expression peaked at 96 h.

### Neuroblastoma proliferation is increased by hypoxia

**Fig. 1:**
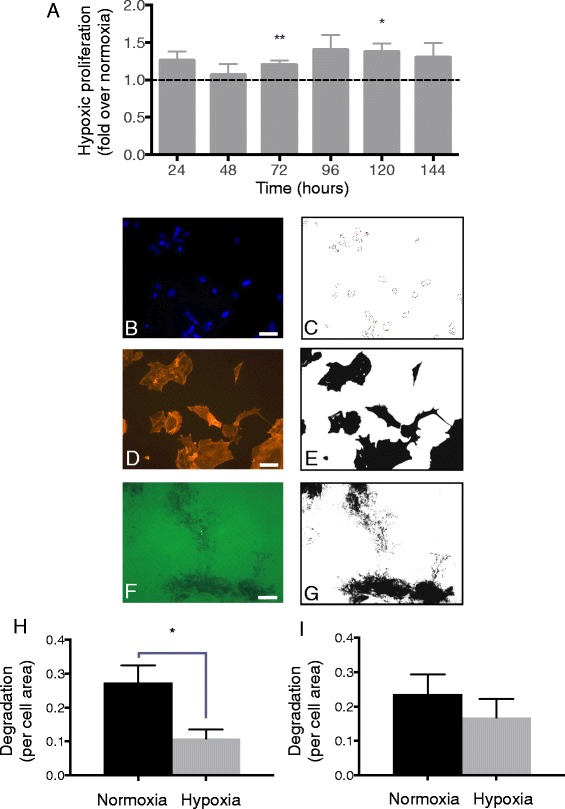
Hypoxia alters the behavior of SH-EP neuroblastoma cells. **a** SH-EP cells were grown in normoxic (20 % O_2_) and hypoxic conditions (1 % O_2_) for 24–144 h. Hypoxic cell viability normalized to normoxic controls (dashed line through 1). **b**-**i** Representative images of a chamber slide (8-well) coated with an extracellular matrix mimic, GFP-tagged gelatin, seeded with 1.6 × 10^4^ SH-EP/well. After 72–96 h ± hypoxia, cells fixed in methanol and stained with DAPI and TRITC-phalloidin. Five fields of view (20× objective) were obtained for each well using a widefield microscope and images imported into Image J for analysis. DAPI nuclear counts (**b**, **c**) provided cell numbers. Thresholding on phalloidin (**d**, **e**) and the absence of GFP (**f**, **g**) provided cell and matrix degradation areas, respectively. Hypoxia resulted in decreased gelatin degradation per cell area at 72 h (**h**), although no significance was observed at 96 h (**i**). 1,000+ cells analysed per timepoint. Data is mean ± SEM; *n* = 4 (**a**), *n* = 3 (**b**-**i**). * *P* < 0.05, ** *P* <0.01 (t-test). Scale bar = 50 μm

Aggressive cancer phenotypes are characterised by increased cellular proliferation. Given that a dynamic actin cytoskeleton is required for proliferation and the transcription factor HIF-2α promotes growth, we investigated the impact of hypoxia on SH-EP cell proliferation. SH-EP were cultured in normoxic and hypoxic conditions for 24–144 h and cell number was determined using trypan blue exclusion. Hypoxic cell counts were significantly increased at 72 and 120 h when normalized to parallel normoxic cell cultures (*P* = 0.007 and *P* = 0.01, respectively; Fig. 1a). Following confirmation of physiological hypoxia at the abovementioned timepoints (8–144 h), we proceeded to examine other aspects of the hypoxic neuroblastoma phenotype.

### Neuroblastoma cell invasiveness is reduced by hypoxia

Cellular invasiveness was measured by a QCM invadopodia assay, which examined degradation of a fluorescent gelatin matrix while controlling for cell number and size. DAPI images (Fig. 1b) were thresholded to provide cell counts (Fig. 1c), while thresholding TRITC-phalloidin images (Fig. 1d) provided cell area values (Fig. 1e). Thresholding GFP images (Fig. 1f) provided matrix degradation areas (Fig. 1g). Hypoxia significantly reduced gelatin degradation per cell area at 72 h (Fig. 1h), though no significant difference was observed at 96 h (Fig. 1i).

### Hypoxia promotes a more organized actin cytoskeleton

**Fig. 2:**
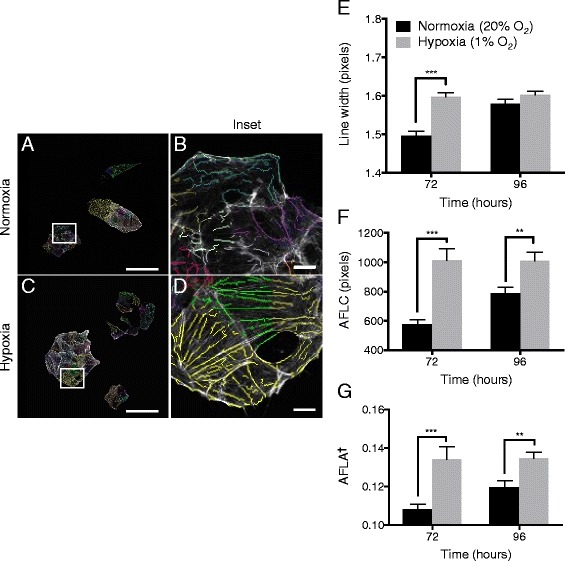
Hypoxia leads to a more ordered actin cytoskeleton. SH-EP cells were grown in normoxia (20 % O_2_) or hypoxia (1 % O_2_) for 72 – 96 h. Coverslips were fixed with 4 % PFA and stained with TRITC-phalloidin and DAPI. Single z-plane images were obtained by confocal microscopy. Linear feature detection software was used to quantitate actin cytoskeletal organization. **a**-**d** Representative linear detection after 72 h ± hypoxia. Hypoxia visibly increases the number of actin filament bundles known as stress fibres (**b** vs. **d**). Scale bar = 50 μm, or 5 μm in inset. **e** Hypoxia increases mean actin filament bundle width at 72 h. **f** Hypoxia increases total actin filament length per cell number (*AFLC*) and (**g**) per cell area (*AFLA*). Data is mean ± SEM (*n* = 3). 650+ cells analysed per timepoint. ** *P* <0.01, *** *P* <0.0001 (t-test). ^†^AFLA is dimensionless, with [length (pixels)]/[area (pixels)]

A dynamic actin cytoskeleton is fundamental to cell migration and invasion. We therefore examined actin cytoskeletal organization to understand the reduction in matrix degradation observed at 72 h hypoxia. Normoxic cells stained with TRITC-phalloidin, which binds to filamentous actin, displayed a disordered actin cytoskeleton (Fig. 2a, b), while hypoxic cells displayed a strong increase in actin cytoskeletal organization (Fig. 2c, d). In particular, hypoxia consistently increased the number of parallel actin filament bundles per cell. The actin-binding protein α-actinin cross-links parallel actin filaments [43], enhances the invasive potential of cancer cells [44] and is associated with poor prognosis in a variety of tumors [45, 46]. For these reasons we examined α-actinin expression as a potential explanation for increased parallel bundles of actin filaments with hypoxia. However, no statistically significant change in expression was observed between normoxic and hypoxic incubations of up to 144 h (data not shown).

Using a linear-feature detection algorithm [42] we quantitated various cytoskeletal parameters, including actin filament bundle width and length (Fig. 2e–g). Mean actin filament bundle width was significantly increased by 72 h hypoxic incubation (Fig. 2e). Actin filament length per cell (AFLC; Fig. 2f) and per cell area (AFLA; Fig. 2g) were significantly increased by 72–96 h hypoxia. The effect of hypoxia on AFLC and AFLA was significant (*P* <0.0001, 2way ANOVA).

### Expression of HMW tropomyosin isoforms, Tm1 and Tm2, are altered by hypoxia

**Fig. 3:**
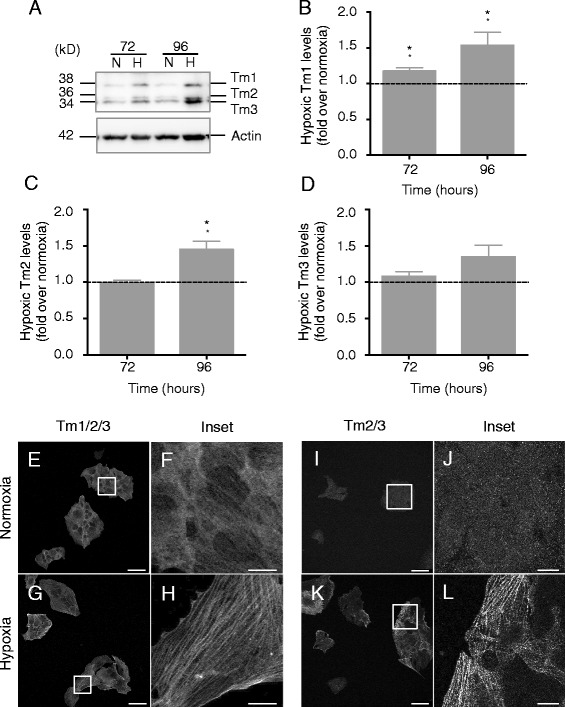
Hypoxia alters the expression and localization of HMW tropomyosins. **a** Normoxic (*N*) and hypoxic (*H*) SH-EP lysates were separated by SDS-PAGE. Representative Western blot stained with α9d antibody, which detects HMW tropomyosin isoforms Tm1, Tm2 and Tm3. Actin used loading control. Densitometry values were normalized to normoxic levels (dashed line) for Tm1 (**b**), Tm2 (**c**) and Tm3 (**d**) expression. Results are mean ± SEM; *n* = 3–4, * *P* <0.05, ** *P* <0.01 (t-test). **e**-**l** SH-EP cells were grown for 72 h ± hypoxia, fixed, permeabilized and incubated with Tm311 and CGβ6 antibodies to detect HMW isoforms Tm1/2/3 (**e**-**h**) and Tm2/3 (**e**-**h**), respectively. Cells then incubated with Alexa555-tagged goat anti-mouse and single z-plane images obtained by confocal microscopy. Hypoxia increased filamentous organization of Tm1/2/3 (**f** vs. **h**) and Tm2/3 (**j** vs. **l**) compared to normoxic control cells. Independent experiments (Tm311, *n* = 2; CGβ6, *n* = 5). Scale bar = 50 μm, 10 μm in inset

Due to the ‘master regulatory’ role of tropomyosins in the actin cytoskeleton [34], changes in tropomyosin expression and localization could provide mechanistic insight into our observed hypoxia-induced reorganization of actin filaments. Clear changes were observed in HMW tropomyosin levels in hypoxic SH-EP cells compared with normoxic controls. HMW isoforms Tm1, Tm2 and Tm3 were detected by immunoblotting with the α/9d antibody (Fig. 3a). Tm1 expression increased significantly over normoxic controls with 72–96 h hypoxia (Fig. 3b), while Tm2 levels increased significantly above normoxia after 96 h hypoxia (Fig. 3c). Although hypoxic Tm3 expression trended upward with time, no statistically significant changes were observed (Fig. 3d).

### Hypoxia increases HMW tropomyosin-containing filaments

Tropomyosins may modulate the actin cytoskeleton in an isoform-specific manner through changes in their intracellular distribution [34]. Cells incubated with antibodies that detect HMW tropomyosin isoforms (Tm1/2/3) displayed an increase in filamentous structures at 72 h hypoxia (Fig. 3g, h) compared with normoxia (Fig. 3e, f). To better discern the role of individual isoforms, we stained for Tm2/3 (CGβ6 antibody). More Tm2/3-containing filaments were observed in hypoxia (Fig. 3k, l) compared to normoxia (Fig. 3i, j).

### Expression and intracellular organization of LMW tropomyosin isoforms are unaltered by hypoxia

**Fig. 4:**
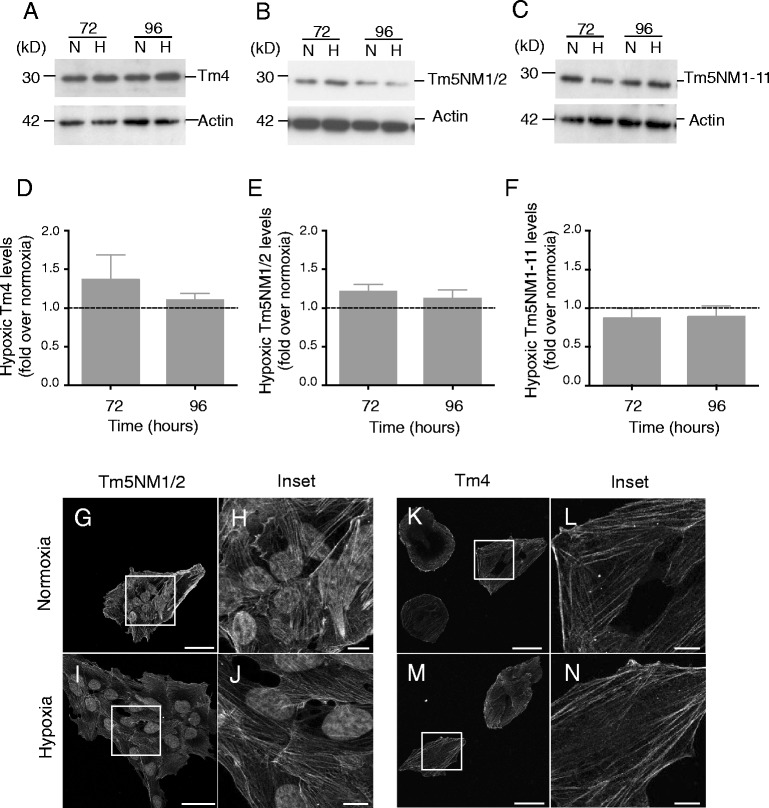
LMW Tropomyosin expression and localization are unaltered by hypoxia. Normoxic (N) and hypoxic (H) SH-EP lysates were separated by SDS-PAGE and immunoblotted with **a** WD4/9d, **b** γ9d and **c** CG3 antibodies to examine expression of LMW isoforms Tm4, Tm5NM1/2 and Tm5NM1-11, respectively. Actin used as loading control (**d**-**f**) Densitometry was normalized to actin, before expressing hypoxic levels relative to normoxic values (*dashed line*). Hypoxia did not significantly alter the expression of these LMW isoforms. Results are mean ± SEM; *n* = 4. **g**-**n** SH-EP cells grown for 72 h ± hypoxia were fixed, permeabilized and incubated with γ9d to detect Tm5NM1/2 (**g**-**j**) and WD4/9d to detect Tm4 (**k**-**n**). Cells then incubated with Alexa555-tagged goat anti-mouse or Alexa488-tagged goat anti-rabbit, respectively. Hypoxia did not alter the intracellular organization of Tm5NM1/2 (**h** vs. **j**) nor Tm4 (**l** vs. **n**). Independent experiments (γ9d, *n* = 2; WD4/9d, *n* = 3). Scale bar = 50 μm, 10 μm in inset

Hypoxia did not induce statistically significant changes in expression of the LMW isoforms Tm4 (Fig. 4a, d), Tm5NM1/2 (Fig. 4b, e), nor Tm5NM1-11 (Fig. 4c, f). Similarly, no hypoxia-induced changes in intracellular organization were observed for Tm5NM1/2 (Fig. 4g–j) and Tm4 (Fig. 4k–n).

## Discussion

Given the essential roles of the actin cytoskeleton in aggressive hypoxic phenotypes, it is surprising that the actin cytoskeleton as a mediator of the hypoxic response has remained largely unexplored until recently [33].

Few studies have examined the effect of hypoxia on neuroblastoma proliferation. However, our observed increase in hypoxic SH-EP proliferation is consistent with a documented marginal increase in SH-EP cell number after 48 h hypoxia [47]. We offer two theories to explain our observation. First, HIF-2α enhances the activity of the oncogenic transcription factor, c-Myc [48], which increases cell proliferation by driving the G_1_/S cell cycle transition [49]. Therefore, hypoxic induction of HIF-2α should promote cell proliferation. Second, hypoxic BE(2)C cells (72 h) induced the expression of both growth factors and growth factor receptors, including VEGF, IGF2, NRP1 and NGFR [16]. Hypoxia might therefore induce autocrine growth and survival loops that drive proliferation.

It is known that 72 h hypoxia induces a ‘fibroblastoid’ phenotype in neoplastic cells of epithelial origin [50]. This suggests involvement of the actin cytoskeleton—a key determinant of cellular morphology—in the hypoxic phenotype. A dynamic actin cytoskeleton is necessary for enhanced migration and invasion [28] and it is likely that our observed hypoxia-induced strengthening of actin cytoskeletal organization—increased actin filament bundle width and length—led to reduced actin dynamics.

Our observation that hypoxia reduced SH-EP cell invasiveness is in contrast to a recent study where hypoxia significantly increased BE(2)C invasion through Matrigel-coated membranes [51]. However, hypoxia was mimicked using deferoxamine mesylate and the invasive phenotype was examined after 24 h hypoxia. Hypoxia also increased neuroblastoma Kelly cell invasion through Matrigel-coated membranes after treating cells to 24–48 h hypoxia [13]. However, these cells were re-oxygenated during the invasion assay. In contrast to SH-EP, which reportedly contain no MYCN mRNA [52], all cell lines displaying increased hypoxic invasiveness were MYCN-amplified. This suggests hypoxia-induced invasion is cell-line-specific and might implicate MYCN status.

The tropomyosin family of actin-associating proteins are known to interact with and regulate actomyosin structures, including actin filament stress fibres. Hypoxia induced the expression of 35 kD and 36.5 kD HMW tropomyosins in porcine pulmonary arterial epithelial cells (PAEC) [53] and it is possible that up-regulated 34 kD and 36 kD proteins in hypoxic human and bovine PAEC were also HMW tropomyosins [54, 55]. However, mammalian evidence of hypoxia-induced tropomyosin synthesis has until now been restricted solely to endothelial cells.

Hypoxia starves cells of energy and a concomitant reduction in protein synthesis is required to maintain essential housekeeping functions. This reversible reduction in overall protein synthesis is HIF-independent [56, 57]. Increased Tm1 and Tm2 levels after 3–4 days hypoxia suggests these proteins must serve a critical, protective role against hypoxic stress, analogous to the induction of heat-shock proteins in hyperthermia [58]. The HMW isoforms Tm1 and Tm2 increase the stability of actin stress fibres [59] and partially protect actin filaments from the severing actions of gelsolin [60, 61]. Moreover, Tm1 and Tm2 display tumor suppressor activity in a range of transformed and tumor cell lines [62–67]. As a dynamic actin cytoskeleton is required for migration and invasion, increased Tm1 and Tm2 expression, and their recruitment to actin filaments, can largely explain the reduced SH-EP invasion at 72 h hypoxia. Presumably, this prevents the energy expenditure associated with actin dynamics. Reduced actin dynamics and induction of tumor suppressor proteins is in stark contrast to the entire notion of an aggressive hypoxia phenotype. It is therefore interesting that expression of these tumor-suppressor isoforms decreased below normoxic levels at 144 h hypoxia (data not shown). We postulate that chronically hypoxic SH-EP might become permissive of the archetypal aggressive phenotype.

The structural and spatial organization of the actin cytoskeleton is highly sensitive to reactive oxygen species (ROS), such as hydrogen peroxide (H_2_O_2_) [68]. In particular, ROS exposure results in a net increase of free barbed ends and a dramatic increase in actin polymerization [69]. More recently, oxidation of non-muscle actin was shown to result in depolymerization of actin filaments and a complete loss of polymerizability [70]. As hypoxia is known to increase intracellular ROS, primarily by the mitochondria [71–73], increased expression of Tm1 and Tm2 may represent an attempt to protect the actin cytoskeleton from oxidative stress. In addition, exposure of human endothelial cells to the ROS, H_2_O_2_, induced ERK-mediated phosphorylation of Tm1, followed by the rapid colocalization with actin and stress fibres [74]. Therefore, the protection afforded by HMW tropomyosins may be a result of increased expression and posttranslational modification that increases their association with the hypoxic actin cytoskeleton.

Altered expression of tropomyosins in hypoxic neuroblastoma cells may involve epigenetic alterations to HMW promoter regions. The TPM1 gene (encoding Tm2) is silenced in metastatic breast and colon cancer cell lines by promoter hyper-methylation. Treatment of these cells with the de-methylating agent 5-aza-2’-deoxycytidine (5-aza-dC) reactivated TPM1 gene expression [75]. Another study demonstrated 5-aza-dC treatment induced up-regulation of TPM2 (encoding Tm1), but not TPM1, in metastatic breast cancer cells [76], while TPM1 and TPM2 were both up-regulated in demethylated fibrosarcoma cells [77]. As hypoxia induces global hypo-methylation [78], we postulate that hypoxia leads to the ‘de-repression’ of tropomyosin expression. Interestingly, most pathways that repress tropomyosin expression affect HMW, but not LMW, tropomyosin isoforms (reviewed in Gunning et al. [34]). HMW isoforms contain the 1a exon, while LMW isoforms contain the 1b exon. As cancer leads to hyper-methylation of CpG islands [78], we performed a qualitative examination of the CpG islands flanking the 1a and 1b exons. This revealed a substantially higher CG content flanking the HMW 1a exon over the LMW 1b exon. This suggests exon 1a-containing HMW tropomyosins may be more susceptible to a hypoxic de-repression of gene expression.

## Conclusions

Hypoxic induction of the tumor-suppressor isoforms, Tm1 and Tm2, might represent an early stress response. Beyond stabilizing the actin filaments, these tropomyosins might protect the actin cytoskeleton from hypoxic stresses that include increased ROS. Changes in actin organization characteristic of reversion of the transformed phenotype are induced by hypoxia at 72–96 h in SH-EP neuroblastoma cells. This is mirrored by increased organization of HMW tropomyosin-containing filaments. However, maximum expression of HMW tropomyosins (96 h) did not correlate with the most significant changes in the cytoskeleton (72 h). Hence, there is no evidence that changes in tropomyosin expression alone can drive the observed cytoskeletal alterations and the increased expression of HMW tropomyosins may be in response to their recruitment into stress fibres. This dissociation of tropomyosin isoform expression and actin organization is intriguing and suggests that other mechanisms may be at work. These findings warrant further investigation into the potential role of tropomyosins in driving hypoxic actin reorganization.

## Additional file

Additional file 1: Figure S1. Hypoxic incubation of SH-EP neuroblastoma cells increases levels of hypoxia-inducible transcription factors, HIF-1α and HIF-2α. SH-EP cells were incubated for 0–144 h in normoxic (20 % O_2_) or hypoxic (1 % O_2_) conditions, before rapidly lysing cells on ice in RIPA buffer containing a protease inhibitor cocktail. Protein lysates were separated using SDS-PAGE and HIFs detected using anti-HIF-1α or anti-HIF-2α antibodies. Representative immunoblots, with HIF expression clearly upregulated in hypoxia (*H*) over normoxic controls (*N*). Cell lysates known to contain HIF-1α and HIF-2α used as positive control (*+ve*). *n* = 3. (PDF 1900 kb)

## Abbreviations

HIF: Hypoxia-inducible factor
HMW: High molecular weight
LMW: Low molecular weight
