# Supplementary figures and images for: Hypoxia alters the recruitment of tropomyosins into the actin stress fibres of neuroblastoma cells
Source: BMC Cancer. 2015 Oct 16;15:712. doi: 10.1186/s12885-015-1741-8 (PMC4608101; doi:10.1186/s12885-015-1741-8)

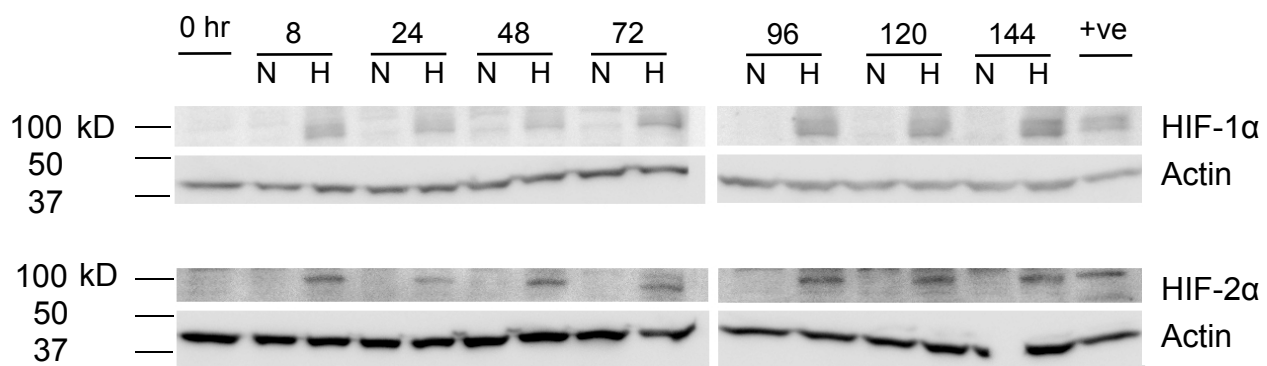

Supplement: Additional file 1: Figure S1. — Hypoxic incubation of SH-EP neuroblastoma cells increases levels of hypoxia-inducible transcription factors, HIF-1α and HIF-2α. SH-EP cells were incubated for 0–144 h in normoxic (20 % O2) or hypoxic (1 % O2) conditions, before rapidly lysing cells on ice in RIPA buffer containing a protease inhibitor cocktail. Protein lysates were separated using SDS-PAGE and HIFs detected using anti-HIF-1α or anti-HIF-2α antibodies. Representative immunoblots, with HIF expression clearly upregulated in hypoxia (H) over normoxic controls (N). Cell lysates known to contain HIF-1α and HIF-2α used as positive control (+ve). n = 3. (PDF 1900 kb) [file 12885_2015_1741_MOESM1_ESM.pdf]
